# Rescue of sharp wave-ripples and prevention of network hyperexcitability in the ventral but not the dorsal hippocampus of a rat model of fragile X syndrome

**DOI:** 10.3389/fncel.2023.1296235

**Published:** 2023-12-01

**Authors:** Leonidas J. Leontiadis, George Trompoukis, Giota Tsotsokou, Athina Miliou, Panagiotis Felemegkas, Costas Papatheodoropoulos

**Affiliations:** Laboratory of Neurophysiology, Department of Medicine, University of Patras, Rion, Greece

**Keywords:** fragile X, neurodevelopmental disorders, hippocampus, dorsoventral, sharp wave-ripple, E-I balance, epileptiform discharges, rat

## Abstract

Fragile X syndrome (FXS) is a genetic neurodevelopmental disorder characterized by intellectual disability and is related to autism. FXS is caused by mutations of the fragile X messenger ribonucleoprotein 1 gene (*Fmr1*) and is associated with alterations in neuronal network excitability in several brain areas including hippocampus. The loss of fragile X protein affects brain oscillations, however, the effects of FXS on hippocampal sharp wave-ripples (SWRs), an endogenous hippocampal pattern contributing to memory consolidation have not been sufficiently clarified. In addition, it is still not known whether dorsal and ventral hippocampus are similarly affected by FXS. We used a *Fmr1* knock-out (KO) rat model of FXS and electrophysiological recordings from the CA1 area of adult rat hippocampal slices to assess spontaneous and evoked neural activity. We find that SWRs and associated multiunit activity are affected in the dorsal but not the ventral KO hippocampus, while complex spike bursts remain normal in both segments of the KO hippocampus. Local network excitability increases in the dorsal KO hippocampus. Furthermore, specifically in the ventral hippocampus of KO rats we found an increased effectiveness of inhibition in suppressing excitation and an upregulation of α1GABA_*A*_ receptor subtype. These changes in the ventral KO hippocampus are accompanied by a striking reduction in its susceptibility to induced epileptiform activity. We propose that the neuronal network specifically in the ventral segment of the hippocampus is reorganized in adult *Fmr1*-KO rats by means of balanced changes between excitability and inhibition to ensure normal generation of SWRs and preventing at the same time derailment of the neural activity toward hyperexcitability.

## 1 Introduction

Fragile X syndrome (FXS) is a neurodevelopmental disorder, the most common inherited form of intellectual disability and the leading genetic cause of autism spectrum disorder (ASD) (Belmonte and Bourgeron, 2006; Hagerman et al., 2017). The primary cause of FXS is the mutation-induced inactivation of *Fmr1* gene leading to the lack of fragile X Messenger Ribonucleoprotein (FMRP) (Verkerk et al., 1991; Bassell and Warren, 2008; Rylaarsdam and Guemez-Gamboa, 2019). FMRP is ubiquitously expressed in the nervous system and is involved in many processes in neuronal cells including the regulation of protein synthesis in axons and dendrites, hence, the loss of FMRP is associated with dysregulation of synaptic and neuronal function (Pfeiffer and Huber, 2007). Phenotypic features of FXS include hyperarousal, hyperactivity, sensory hypersensitivity, learning and memory deficits, anxiety, seizures, social deficits, and disturbances in information processing (Hagerman, 2006; Kidd et al., 2014).

Fragile X syndrome affects various brain regions including the hippocampus (Hessl et al., 2004; Molnár and Kéri, 2014), thereby affecting functions such as memory consolidation (Gatto and Broadie, 2009) and learning flexibility (Cell, 1994; D’Hooge et al., 1997) that require normal function of the hippocampus (Nadel and Moscovitch, 1997; Watson and Stanton, 2009; Vilá-Balló et al., 2017). Hippocampus-dependent memory consolidation involves the neuronal network activity of sharp wave-ripples (SWRs) (Girardeau and Zugaro, 2011; van de Ven et al., 2016), an endogenous network oscillation of the hippocampus (Buzsaki, 2015). Evidence demonstrates that memory consolidation and SWRs occur primarily during sleep (Wilson and McNaughton, 1994; Jadhav et al., 2012; Brodt et al., 2023), which appear to be disrupted in FXS (Saré et al., 2017; Boone et al., 2018; Martinez et al., 2023). SWRs are local field potentials intrinsically generated by the hippocampal circuitry and consist of a slow potential (i.e., the slow wave) ridden by high-frequency oscillation (ripple, 100–200 Hz). Intense multiunit activity during SWRs represents the highly synchronous firing of pyramidal cells and interneurons which gives rise to the ripple oscillation (Ramirez-Villegas et al., 2018). In addition to memory consolidation, SWRs are also implicated in stress/anxiety (Tomar et al., 2021; Kuga et al., 2023), which have been suggested to be affected in FXS (Crawford, 2023).

Oscillations of neural circuits are fundamental expressions of brain activity (Buzsaki, 2006), and are critically regulated by a dynamic excitation and inhibition (E-I) balance (Mehta et al., 2010). Persistent alterations in E-I balance may lead to disruption of network oscillations leading to cognitive impairments (Cherubini et al., 2021). Abnormalities in brain oscillations including SWRs have been reported to occur in neuropsychiatric disorders including schizophrenia (Suh et al., 2013; Gao et al., 2019), Rett syndrome (D’Cruz et al., 2010), Down syndrome (Alemany-González et al., 2020), and anxiety disorders (Caliskan and Stork, 2019). Furthermore, FXS is accompanied by changes in gamma and theta oscillations (Radwan et al., 2016; Wang et al., 2017; Arbab et al., 2018), and alterations in gamma oscillation and SWRs have been recently detected in a Cntnap2 mouse model of autism (Paterno et al., 2021). Although there are some recent data suggesting that SWRs are altered in FXS (Boone et al., 2018; Pollali et al., 2021), the relationships between SWRs and FXS are insufficiently investigated and possible underlying mechanisms of SWR alterations in FXS remain elusive.

It is widely accepted that FXS and other neurodevelopmental disorders are mechanistically linked to a disturbance in the balance between excitation and inhibition (E-I) toward excitation in several brain regions of individuals and animal models of FXS (Nelson and Valakh, 2015; Sohal and Rubenstein, 2019). Increase of E-I ratio in FXS may result from an increased intrinsic cellular excitability and/or a reduction in synaptic inhibition (Contractor et al., 2015; Nelson and Valakh, 2015; Liu et al., 2021; Nomura, 2021; Bülow et al., 2022). A strong consensus points to a decline in several aspects of GABAergic inhibition in FXS, see reviews by Paluszkiewicz et al. (2011), Filice et al. (2020), Van der Aa and Kooy (2020), and Nomura (2021) and manipulations that enhance GABAergic inhibition can alleviate several behavioral deficits in animal models of FXS and autism (Olmos-Serrano et al., 2011; Selimbeyoglu et al., 2017; Goel et al., 2018) as well as in experimental models of elevated cortical E-I balance (Yizhar et al., 2011); see reviews by D’Hulst and Kooy (2007), Cellot and Cherubini (2014), Lozano et al. (2014), and Braat and Kooy (2015). Paradoxically, however, pharmacological treatment that enhances GABAergic transmission has not yet yielded clearly positive effects in patients with FXS (Ligsay et al., 2017; Van der Aa and Kooy, 2020); furthermore, some aspects of GABAergic inhibition are enhanced, instead of reduced, in the *Fmr1*-KO mice (Cea-Del Rio et al., 2020; Yang et al., 2020).

Normal generation of SWRs requires a balance between excitation and inhibition (Buzsaki, 2015; Melonakos et al., 2019). Therefore, changes in the E-I balance that are suggested to occur in neurodevelopmental disorders (Gao and Penzes, 2015; Kenny et al., 2022) may influence physiological generation of SWRs. Interestingly, previously accumulated evidence shows that FXS-associated neurobiological changes are brain region-specific (Anagnostou and Taylor, 2011; Varghese et al., 2017; Fetit et al., 2021) and hippocampus is among the brain regions that are affected by the loss of FMRP (Banker et al., 2021; Liu et al., 2022). However, the hippocampus is a functionally heterogeneous structure in both health and disease (Bannerman et al., 2014; Strange et al., 2014) and the dorsal and the ventral hippocampus are differently implicated in neuropsychiatric and neurodevelopmental disorders; reviewed by Sahay and Hen (2007), Tanti and Belzung (2013), Bartsch and Wulff (2015), Gulyaeva (2019), and Bakoyiannis et al. (2023). For example, the effects of chronic antidepressant treatment are specifically mediated through the anterior or ventral hippocampus in human or rodents, respectively (Banasr et al., 2006; Sahay and Hen, 2007; Boldrini et al., 2009) and schizophrenia affects mainly the anterior hippocampus (Szeszko et al., 2002). Interestingly, the dendritic spine density is oppositely affected in the dorsal and ventral hippocampus in the valproic acid animal model of autism (Bringas et al., 2013) and there is a septotemporal variation in the processing of social information (Watarai et al., 2021) which is disrupted in FXS. However, it is not yet known whether FXS differentially affects neuronal activity along the hippocampal long axis.

Here, using transverse hippocampal slices from adult rats and recordings of field potentials we show that SWRs and associated multiunit activity are altered in parallel with increased local network excitability in the dorsal hippocampus of *Fmr1*-KO rats. In contrast, normal SWRs and firing activity were observed in the ventral KO hippocampus which is endowed with increased GABAergic inhibition and a striking resistance to induced epileptiform activity. Our results show that the dorsal and ventral hippocampus respond unequally to the loss of FMRP suggesting that some changes occurring in the brain of subjects suffering from neurodevelopmental disorders may represent the outcome of homeostatic processes that attempt to keep neuronal network function effective.

## 2 Materials and methods

### 2.1 Animals and hippocampal slices

Long Evans (LE) male rats 3–4 months old were used in this study. Both wild type (WT) and *Fmr1*-KO (KO) LE rats were purchased from Medical College of Wisconsin (RRIDs: RGD_ 2308852 and RGD_ 11553873, respectively). Rats were maintained under stable conditions of light-dark cycle (12/12 h), temperature (20–22°C) and they had free access to food and water, in the pathogen-free Laboratory of Experimental Animals of the Department of Medicine of the University of Patras (license No: EL-13-BIOexp-04). The treatment of animals and all experimental procedures used in this study were conducted in accordance with the European Communities Council Directive Guidelines for the care and use of Laboratory animals (2010/63/EU–European Commission) and approved by the Protocol Evaluation Committee of the Department of Medicine of the University of Patras and the Directorate of Veterinary Services of the Achaia Prefecture of Western Greece Region (reg. number: 5661/37, 18/01/2021). In addition, this animal study was reviewed and approved by the Research Ethics Committee of the University of Patras. Rats were genotyped using tail or brain tissue to test the expression of FMRP by means of Western blotting.

We prepared slices from both the dorsal and the ventral hippocampus of WT and KO rats. Specifically, we decapitated an individual rat under conditions of deep anesthesia with diethyl-ether (ChemLab NV, Belgium) using a home-made guillotine; then, the brain was removed from the cranium and placed in ice-cold (2–4°C) standard medium containing, in mM: 124 NaCl, 4 KCl, 2 CaCl_2_, 2 MgSO_4_, 26 NaHCO_3_, 1.25 NaH_2_PO_4_, and 10 glucose. The medium was equilibrated with 95% O_2_ and 5% CO2 gas mixture at a pH = 7.4. Hippocampi were removed from the two hemispheres and placed on the plate of a McIlwain tissue chopper. Then, by cutting hippocampus transversely to its long axis we prepared 500–550 μm thick slices from the two opposite segments of the hippocampus, dorsal and ventral. Specifically, we used the tissue extending between 0.5 and 3.5 mm from each end of the structure. Immediately after their preparation, hippocampal slices were placed to a home-made plexiglass interface type (air-liquid) recording chamber. Slices in the recording chamber were maintained at a constant temperature of 30 ± 0.5°C, and they were continuously perfused with standard medium of the same composition as described above and at a perfusion rate of ∼1.5 ml/min. Slices were constantly humidified with a mixed gas consisting of 95% O2 and 5% CO2. The chamber consisted of two independent compartments in each of which we placed about ten hippocampal slices. Slices prepared from a given hippocampus were placed in both compartments. Slices were examined alternately between the two compartments such that slices from both hippocampal segments and from both genotypes were studied at similar times from their placement in the chamber. Tissue temperature was continuously monitored and regulated via a heated water tank beneath the two compartments. To ensure a steady flow of artificial cerebrospinal fluid, we used a home-made gravity perfusion system with continuous monitoring of liquid flow. The slices were left for at least one and a half hours to recover, and then stimulation and recording were started.

### 2.2 Electrophysiology

Spontaneous and evoked field potentials were recorded from the stratum radiatum and the stratum pyramidale of the CA1 hippocampal field, where the apical dendrites and the somata of pyramidal cells are located, respectively. Occasionally, simultaneous recordings from the CA3 and the CA1 pyramidal layer were done using electrode pairs. Recordings were done in the middle proximal-distal position using carbon fiber electrodes 7 μm-thick (Kation Scientific, Minneapolis, MN, USA). Evoked field potentials were recorded following electrical stimulation of Schaffer collaterals, i.e., the axons of CA3 pyramidal cells that synapse onto the dendrites of CA1 neurons. Electrical stimulation consisted of constant current pulses with a stable duration of 100 μs and variable amplitude. Stimulation current was delivered using a home-made bipolar platinum/iridium wire electrode with a wire diameter of 25 μm and an inter-wire distance of 100 μm; wire was purchased from World Precision Instruments, USA. Stimulation and recording electrodes were placed in slices under visual guidance using three-axis mechanical micromanipulators (Narishige Group, Japan) and a stereo microscope (Olympus, Japan) under fiber optic lighting (Volpi AG, USA). Signal was acquired and amplified X500 and then filtered at 0.5 –2 kHz using Neurolog systems (Digitimer Ltd, UK), consisting of AC preamplifier (NL 104A with NL 100AK headstage), AC/DC amplifier (NL 106) and band pass filter (NL 125/6). Analog signal was digitized at 10 kHz using a CED 1401-plus interface and the Spike or Signal software (Cambridge Electronic Design, Cambridge, UK), then, stored on a computer disk for off-line analysis using the same softwares. Also, signal was continuously optically monitored using an analog-digital oscilloscope (Hameg Instruments, Germany). We also used a Neurolog audio amplifier (NL 120S) for audio monitoring the signal. Stimulation current pulses were delivered using a DS3 constant current stimulator (Digitimer Ltd, UK).

#### 2.2.1 Spontaneous potentials

**FIGURE 1:**
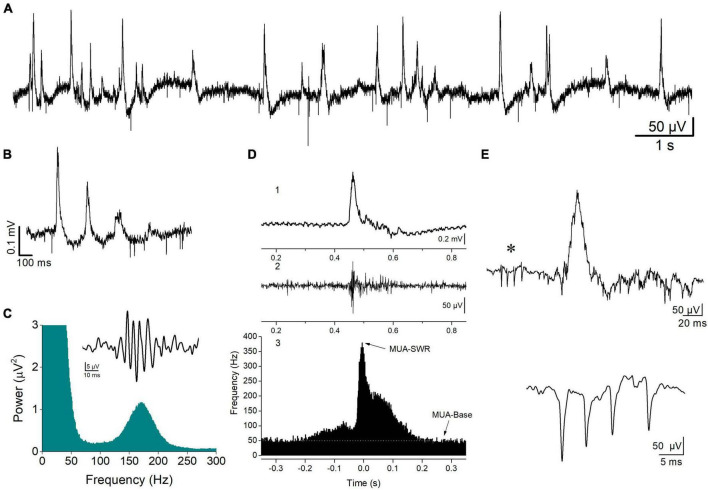
Spontaneous activity recorded from the stratum pyramidale of CA1 hippocampal field. **(A)** A record at slow speed showing sharp wave—ripples (SWRs, positive-going potentials) and unit activity (downward deflections) occurring in a hippocampal slice from a WT rat. SWRs occur either isolated or in sequences of multiple events, i.e., clusters **(B)**. An example of a cluster of SWRs is shown. **(C)** FFT showing the high frequency peak at 150–200 Hz produced by the ripple oscillation. Insert shows a single ripple as revealed by band-pass filtering at 90–300 Hz. **(D)** A single event of SWR (1), the multiunit activity (MUA) revealed after band-pass filtering raw record at 0.4–1.5 kHz (2), and the peri-event histogram of MUA triggered by the peaks of sharp waves (3). Arrows show the locations of the distribution histogram used to measure the maximum frequency of MUA during SWR (MUA-SWR) and the baseline MUA (MUA-Base, white line). **(E)** A complex spike burst (CSB, asterisk) preceding a single SWR event (upper trace), and an example of CSB recorded from a different hippocampal slice (lower trace) are shown.

Spontaneous field potentials were recorded from the CA1 pyramidal layer and consisted of physiological and epileptiform activity. Physiological activity consisted of complexes of sharp waves–ripples (SWRs), multiunit activity, and activity from identified single units (single-unit activity) (Figure 1). Events of SWRs occurred either isolated or in characteristic sequences of two or more consecutive events called clusters (Figures 1A, B), which displayed a stereotyped interval between consecutive events, the intra-cluster interval, ICI. Both isolated single events and clustered events are called episodes. Clusters were detected by the short and markedly stable interval between consecutive events inside a cluster (intra-cluster interval, ICI, ∼100 ms) reflected in distribution histogram of the IEI. In these distribution histograms we could determine the range of relatively short intervals which corresponded to ICI. To measure the various parameters of events of SWRs original records were down sampled (at 1 kHz) and low-pass filtered at 35 Hz. Then, individual events were detected after setting a threshold at a level where all putative events were identified as verified by visual inspection as previously described (Giannopoulos and Papatheodoropoulos, 2013). As previously described (Kouvaros and Papatheodoropoulos, 2017; Trompoukis et al., 2020; Caliskan et al., 2023), events of SWRs were quantified by (1) the amplitude of the SPWs measured by the voltage difference between the positive peak and the baseline. (2) The inter-event interval (IEI) measured as the time between successive individual SWRs. (3) The probability of occurrence of clusters measured as the number of clusters divided by the number of all episodes. (4) The probabilities of clusters with more than two events (“long-clusters”). (5) The ICI measured as the mean value of the intervals between consecutive events inside a cluster. (6) The CA1-CA1 auto-corelation, quantified by the value of the second positive peak in auto-correlograms. (7) The CA3-CA1 cross-correlation, quantified by the value of the first positive peak in cross-correlograms. (8) The power of the ripple oscillation. (9) The frequency of the ripple oscillation. Because complex SWR activity are discrete events, their rate of occurrence can be quantified by measuring the IEI, while the peak frequency as well as the power of the ripple oscillation is reliably quantified from power spectra graphs that displayed the coefficients for each frequency measured by the fast Fourier transform (FFT) (Figure 1C). Clustered SWRs represent a typical pattern of occurrence of SWRs in the intact hippocampus (Buzsaki, 2015), which displays specific properties including the short and relatively stable interval between consecutive events and their dependence on NMDA receptors (NMDARs) (Papatheodoropoulos, 2010). Furthermore, the probability of occurrence/length of clustered SWRs and the ICI are sensitive to the level of GABAergic transmission (Koniaris et al., 2011; Papatheodoropoulos and Koniaris, 2011; Giannopoulos and Papatheodoropoulos, 2013) or the activity of ion channels (Trompoukis et al., 2020), which might be altered in the FXS hippocampus. Autocorrelation was used to measure the degree of rhythmicity (Steriade et al., 1991) of local SWRs in CA1 field. Cross-correlation was used as an index of waveform similarity and spatial coherence (Gafurov and Bausch, 2013; Caliskan et al., 2023) of sharp waves between the CA3 and CA1 hippocampal fields. The amplitude, IEI, autocorrelation, and cross-correlation were measured in low-pass (<35 Hz) filtered records. The probability of clusters of SWRs was calculated using raw records. The amplitude, IEI and ICI of SWRs were measured from 5-min-long raw records, which were also used to prepare power spectra graphs. The probability of clusters was calculated from a 1-min-long period randomly selected from a 5-min-long record.

Multiunit activity (MUA), which quantifies the degree of neuronal excitation (Mölle et al., 2006), was revealed in band-pass filtered records (at 400–1.5 kHz) and was detected by setting a threshold level at a value that all putative events (i.e., negative spikes) were identified as verified by visual inspection, as previously described (Kouvaros and Papatheodoropoulos, 2017). MUA that occurred between events of SWRs is called MUA-Base, and during SWRs is called MUA-SWR. We quantified both MUA-Base and MUA-SWR by its frequency of occurrence (Hz). MUA-Base was measured by the frequency of MUA at steady state between consecutive events of SWRs. We measured MUA-SWR by the maximum frequency of MUA in peri-event histograms between SWRs and MUA, where we used the positive peaks of low-pass filtered SWRs as reference events (Figure 1D). The peak of MUA during SWRs is in phase with ripple oscillation (Chrobak and Buzsaki, 1996), however, it precedes the peak of the slow sharp wave by ∼5 ms (Kouvaros and Papatheodoropoulos, 2017). Thus, we measured the delay between MUA and SWRs and we call the corresponding variable MUA-Delay.

Single-unit activity consisted of isolated bursts of two or more spikes that could be detected between episodes of SWRs, which are typically observed *in vivo* and are called complex spikes (CSB) (Fox and Ranck, 1981; Suzuki and Smith, 1985; Figure 1E). Therefore, we detected and quantified single-unit bursts recorded *in vitro* as previously described (Kouvaros and Papatheodoropoulos, 2017), using previously proposed criteria for the analysis of CSB in the hippocampus (Ranck, 1973; Fox and Ranck, 1981). Specifically, we used the following criteria to detect and quantify CSB: (a) the CSB composed of two or more spikes; (b) the amplitude of consecutive spikes in CBS usually declined from the first to the last; and (c) the interval between consecutive spikes, i.e., the inter-spike interval ranged from about 2 to 12 ms. We also used additional criteria to identify putatively distinct units, including the shape and amplitude of the first spike in a burst. Auxiliary, we also considered the stability of the number of spikes per CSB over time since this has been shown to represent a property of CSB (Suzuki and Smith, 1985). When following the above criteria and encountered difficulty to perform a segregation of CSB into different individual units, we assumed that the different CBS were elicited by a single unit. Measures of complex spike bursts were made from records between events of SWRs. A total period of at least 10 min was used to detect and quantify CSB. We quantified CSB by the number of spikes per burst, and the mean inter-spike interval (inter-spike interval, ISI).

Spontaneous population discharges resembling interictal epileptiform discharges were induced by removing magnesium ions (Mg^2+^) from the perfusion medium (i.e., Mg^2+^-free medium). Hippocampal slices were perfused with Mg^2+^-free medium at about 1 h after tissue was placed in the recording chamber. Population discharges started to occur spontaneously in both dorsal and ventral hippocampal slices as previously observed (Papatheodoropoulos et al., 2005). Spontaneous interictal-like discharges induced by Mg^2+^-free medium are thought to reflect large depolarizations produced mainly by activation of NMDARs (Dingledine et al., 1986) because of receptor relief from Mg^2+^-mediated blockade (Ascher and Nowak, 1988). Interictal-like population discharges were also induced following blockade of GABA_*A*_ receptors (GABA_*A*_Rs) by 50 μM picrotoxin (PTX) and are called disinhibition-induced discharges.

#### 2.2.2 Evoked potentials

Evoked extracellular potentials consisted of excitatory postsynaptic potentials (fEPSPs) and population spikes (PSs) were recorded from the stratum radiatum and stratum pyramidale, respectively. fEPSP was quantified by the maximum slope (fEPSP slope) of the early rising phase (Andersen et al., 1980a). PS was quantified by its amplitude measured as the length of the projection of the minimum peak on the line connecting the two maximum peaks of the PS waveform (Andersen et al., 1980b). Input-output curves between stimulation current intensity and fEPSP slope or PS were systematically constructed in each slice. In the corresponding graphs, stimulation current intensity was normalized with respect to the maximum current intensity used in a particular slice.

The relationship between the stimulation current intensity and fEPSP slope was used to estimate synaptic effectiveness while the relationship between stimulation current intensity and PS was used to estimate neuronal excitation. Local network excitability was assessed by the relationship between synaptic depolarization (fEPSP slope) and neuronal firing (PS), i.e., the PS/fEPSP slope ratio (Andersen et al., 1980b; King et al., 1985). The strength of feedback synaptic inhibition in the local network of the CA1 field was assessed by using the paired-pulse stimulation paradigm and recordings of PS (Andersen and Lomo, 1969; Lee et al., 1979; Ashton and Wauquier, 1985; Papatheodoropoulos and Kostopoulos, 1998; Sharvit et al., 2015). According to this stimulation protocol two identical pulses are applied in rapid succession at the Schaffer collaterals; the excitation of pyramidal cell elicited by the first pulse (PS1) leads to activation of a local network of inhibitory neurons which suppress firing of pyramidal cells evoked by the second pulse (PS2) (Spencer and Kandel, 1961; Andersen et al., 1964), via activation of GABA_*A*_R (MacIver, 2014). We quantified the so-produced paired-pulse inhibition (PPI) by the PS2/PS1 ratio, and the potency of PPI is expressed by a reduction in this ratio.

### 2.3 Western blotting

#### 2.3.1 FMRP protein detection

Following the excision of the hippocampus, parts of the remaining brain tissue were stored at −80°C for a post-mortem protein expression analysis. Later, 20–40 mg tissue samples from various rats were solubilized in 200–400 μL of lysis buffer containing 1% sodium dodecyl sulfate (SDS) and protease inhibitors at a 1:100 dilution and homogenized with sonication. Alternatively, the tip of the tail of a living rat was solubilized in 200 μL of lysis buffer and homogenized, as described above, in the case of ante-mortem protein expression analysis. The protein concentration for each brain or tail extract was determined by using the NanoDrop 2000 Spectrophotometer (Thermo Scientific, Waltham, MA, USA). A 40–50 μg electrophoresis sample was generated from each protein sample by adding 5x sample buffer to the appropriate protein sample volume, followed by 5 min boiling. Proteins were separated by SDS poly-acrylamide gel electrophoresis (SDS-PAGE) and transferred to a polyvinylidene difluoride membrane (Amersham Hybond-P Western blotting PVDF membrane, Sigma, GE10600029) by Western blotting. After 1 h of blocking at room temperature (RT) in a phosphate buffered saline containing 0.1% Tween 20 (PBST) and 5% non-fat dried milk, the PVDF membrane was incubated at 4°C overnight with a rabbit anti-FMRP polyclonal antibody (1:1,500 dilution, Abcam, 17722). The blot was rinsed 3 times for 5 min with PBST and then incubated with goat anti-rabbit horseradish peroxidase (HRP)-linked secondary antibody (1:3,000 dilution, Cell Signaling, #7074) for 1 h at RT. Both antibodies were diluted in PBST containing 3% non-fat dried milk. Immunodetection was carried out using an Enhanced Chemiluminescence detection system (Pierce ECL Western Blotting Substrate, Thermo Scientific, 32209) per the manufacturer’s instructions. Chemiluminescence from the blots was detected by exposing the membranes to X-ray film (Super RX-N, Fujifilm, 47410-19289) for 20 s to 5 min and FMRP expression was confirmed by the detection of a protein band at 75–80 kDa.

#### 2.3.2 α1 GABA_*A*_R and NMDARs

The CA1 region of KO and WT from both dorsal and ventral hippocampus was homogenized with sonication in 200 μL of 1% SDS containing protease inhibitors at 1:100 dilution. The homogenates were stored at −80°C. Protein concentration was determined for each sample using the NanoDropTM 2000/2000c Spectrophotometers (Thermo Scientific). CA1 region of dorsal and ventral hippocampus homogenate (25 μg of protein per lane) were subjected to SDS-PAGE on 10% polyacrylamide gels for 30 min at 80 V followed by 1 h at 120 V. Proteins were transferred to PVDF membrane at 400 mA for 90 min. To block non-specific sites, membranes were incubated for 1 h at RT in 5% non-fat dried milk in PBST. Membranes were next incubated overnight at 4°C with the following primary antibodies diluted in 3% PBST: rabbit anti-α1 GABA_*A*_R polyclonal antibody (1:2500 #06-868, Millipore Sigma), rabbit anti-NR1 monoclonal antibody (1:1000 #D65B7, Cell Signaling), rabbit anti-NR2A polyclonal antibody (1:1000 #4205, Cell Signaling), rabbit anti-NR2B monoclonal antibody (1:1000 #B8E10, Cell Signaling) and rabbit anti-β-actin polyclonal antibody (1:15000 #E-AB-20058, Elabscience). The blots were rinsed with PBST and then incubated with secondary HRP-conjugated goat anti-rabbit IgG antibody (1:8000-1:15000 or 1:60000 #AP132P, Merck Millipore) for 60 min at RT. Molecular masses were determined by comparison with prestained protein molecular weight marker standards (27–200 kDa) (#MWP03, Nippon Genetics). The bands were visualized on ChemiDoc MP (BioRad) by enhanced chemiluminescence (Immobilon ECL Ultra Western HRP Substrate, # WBULS0500, Millipore) for 1 to 10 min. Densitometric quantification of immunopositive bands was carried out. Optical density measurements from each band were defined as ROD units with ImageLab 6.1. The ROD of each band was quantified relatively to the ROD of β-actin which serves as a gel loading control. Then, the ratio, (ROD of protein of interest)/(ROD β-actin) was normalized with the same ratio of a sample used as an internal control, which was loaded in all gels.

### 2.4 Drugs

The following drugs were used: the antagonist of ionotropic non-NMDARs 6-Cyano-7-nitroquinoxaline-2,3-dione disodium salt (CNQX, 40 μM), the selective antagonist of NMDARs 3-[(R)-2-Carboxypiperazin-4-yl]-propyl-1-phosphonic acid (CPP, 10 μM ), the antagonist of GABA_*A*_Rs SR 95531 (gabazine, 5 μM), and the blocker of GABA_*A*_R picrotoxin (PTX, 50 μM ). CNQX and gabazine were purchased from Tocris Cookson Ltd, UK; CPP was purchased from Sant Cruz, CA, USA; and PTX was obtained from Sigma-Aldrich, Germany. Drugs were first prepared as stock solutions and then dissolved in standard medium, and bath applied to the tissue. Stock solutions were prepared in distilled water.

### 2.5 Statistics

In this study, rat was the experimental unit, and the statistics were performed using the number of rats (except when otherwise indicated). However, correlations between variables and comparisons of disinhibition-induced discharges were performed using the number of slices. Furthermore, statistics on complex spike bursts were made using the number of identified putative units. This is specified in the relevant text. The univariate full factorial or univariate multifactorial general linear model (UNIANOVA) with two fixed-effect factors and the parametric independent *t*-test (excluding cases analysis by analysis) were used to assess the genotype or region effects on the various parameters. The two-tailed bivariate correlation analysis (excluding cases pairwise) was used to assess the degree of correlation between parameters. The IBM SPSS Statistics 27 software package was used for all statistical analyses. Collective data in figures are presented by box and whisker plots showing the median with the 25th and 75th quartiles (diamond box), the mean and the 5th and 95th percentile (thick line through small box and whiskers, respectively), individual data points and the normal distribution curve. Values in Tables represent mean ± S.E.M.

## 3 Results

### 3.1 SWRs and MUA differ between the dorsal and ventral hippocampus in WT rats

Spontaneous SWRs were recorded from dorsal and ventral hippocampal slices obtained from WT and KO rats. First, we compared all types of spontaneous activity between the dorsal and ventral hippocampus of WT rats, and we found that SWRs and MUA differ between the two segments of the hippocampus in the WT rat. Specifically, the ventral compared with the dorsal hippocampus displayed significantly higher amplitude of SWRs, lower probability of occurrence of clusters of SWRs, lower probability of long clusters (clusters with more than 2 events) of SWRs, and higher CA1-CA1 autocorrelation. Furthermore, the frequency of MUA during SWRs has been found increased in the ventral compared with the dorsal hippocampus. No significant dorso-ventral differences in the other parameters were observed. Statistics about comparisons of spontaneous activities between dorsal and ventral hippocampus in WT rats are provided in Table 1.

**TABLE 1:** Comparisons of spontaneous activities between the dorsal and ventral hippocampus of WT rats.

		Dorsal hippocampus	Ventral hippocampus	Independent *t*-test
SWRs	Amplitude (μV)	78.5 ± 8.2 (17)	125.7 ± 10.84 (26)	*t*_41_ = −3.15, *p* = 0.003
	IEI (s)	0.991 ± 0.205 (17)	0.72 ± 0.05 (26)	*t*_17.76_ = 1.26, *p* = 0.223
	Probability clusters	0.236 ± 0.033 (12)	0.05 ± 0.01 (20)	*t*_30_ = 5.2, *p* < 0.001
	Probability long clusters	0.195 ± 0.033 (12)	0.05 ± 0.03 (20)	*t*_30_ = 3.22, *p* = 0.003
	ICI (ms)	114 ± 12.3 (10)	144 ± 18.6 (12)	*t*_20_ = −1.26, *p* = 0.221
	Auto-correlation CA1-CA1	0.015 ± 0.0095 (11)	0.17 ± 0.024 (14)	*t*_16.98_ = −6.11, *p* < 0.001
	Cross-correlation CA3-CA1	0.65 ± 0.032 (4)	0.731 ± 0.036 (14)	*t*_16_ = −1.13, *p* = 0.275
Ripples	Power (mV × 10^–7^)	0.444 ± 0.121 (6)	1.54 ± 0.483 (18)	*t*_22_ = −1.29, *p* = 0.211
	Frequency (Hz)	168.5 ± 11.35 (6)	171.33 ± 3.24 (18)	*t*_5.835_ = −0.24, *p* = 0.819
MUA	MUA Baseline	20.46 ± 4.75 (11)	25.23 ± 4.19 (14)	*t*_23_ = −0.754, *p* = 0.459
	MUA–SWR	190.8 ± 28.97 (11)	352.32 ± 46.88 (14)	*t*_20.87_ = −2.93, *p* = 0.008
	MUA–Delay	4.52 ± 0.50 (11)	5.73 ± 0.61 (14)	*t*_23_ = −1.47, *p* = 0.156
CSB	No of spikes	2.44 ± 0.18 (16)	2.19 ± 0.11 (12)	*t*_26_ = 1.09, *p* = 0.285
	ISI (ms)	7.22 ± 0.36 (16)	6.8 ± 0.26 (12)	*t*_26_ = 0.897, *p* = 0.378

Values represent mean ± S.E.M.

In parenthesis is shown the number of rats used in the analysis for all variables except the variables of CSB where the number of units is shown.

### 3.2 SWRs are altered in the dorsal hippocampus but remained normal in the ventral KO hippocampus

**FIGURE 2:**
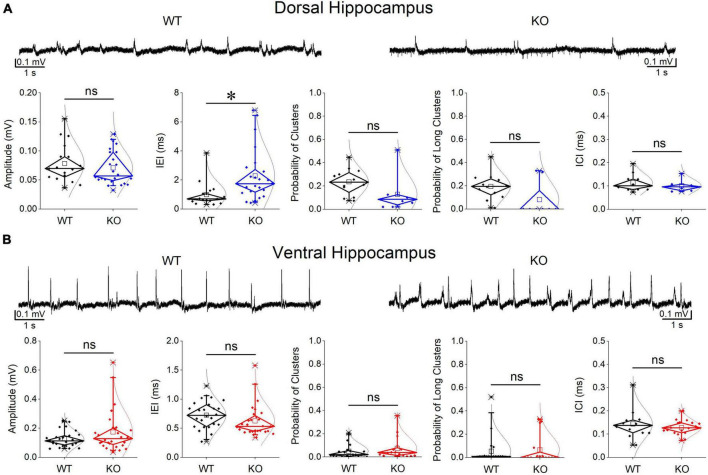
Comparison of SWRs between WT and KO rats. Genotype affects SWRs in the dorsal **(A)** but not the ventral hippocampus **(B)** of KO rats. Data for the amplitude of SWRs’ events, the inter-event interval (IEI), the probability of appearance of clusters of SWRs, and the intra-cluster interval (ICI) are shown. Examples of records are shown at the top of the graphs. The results of statistical analysis (independent *t*-test) for the four variables are the following: amplitude of SWRs: dorsal hippocampus *t*_39_ = 0.764, *p* = 0.45, WT = 17 rats, KO = 24 rats, and ventral hippocampus *t*_35_._23_ = –1.64, *p* = 0.110, WT = 26 rats, KO = 28 rats; IEI: dorsal hippocampus *t*_34.88_ = –3.102, *p* = 0.004, WT = 17 rats, KO = 24 rats, and ventral hippocampus *t*_52_ = 1.39, *p* = 0.17, WT = 26 rats, KO = 28 rats; probability of clusters: dorsal hippocampus *t*_18_ = 1.77, *p* = 0.094, WT = 12 rats, KO = 8 rats, and ventral hippocampus *t*_34_ = –0.905, *p* = 0.372, WT = 20 rats, KO = 16 rats; probability of long clusters: dorsal hippocampus *t*-test, *t*_18_ = 1.94, *p* = 0.069, WT = 12 rats, KO = 8 rats, and ventral hippocampus *t*_34_ = –0.299, *p* = 0.767, WT = 20 rats, KO = 16 rats; ICI: dorsal hippocampus *t*_28_ = 0.874, *p* = 0.39, WT = 10 rats, KO = 6 rats, and ventral hippocampus ICI (*t*_14_ = 0.692, *p* = 0.50, WT = 12 rats, KO = 18 rats. Asterisk and “ns” in this and following figures denote statistically significant and not significant difference, respectively.

**FIGURE 3:**
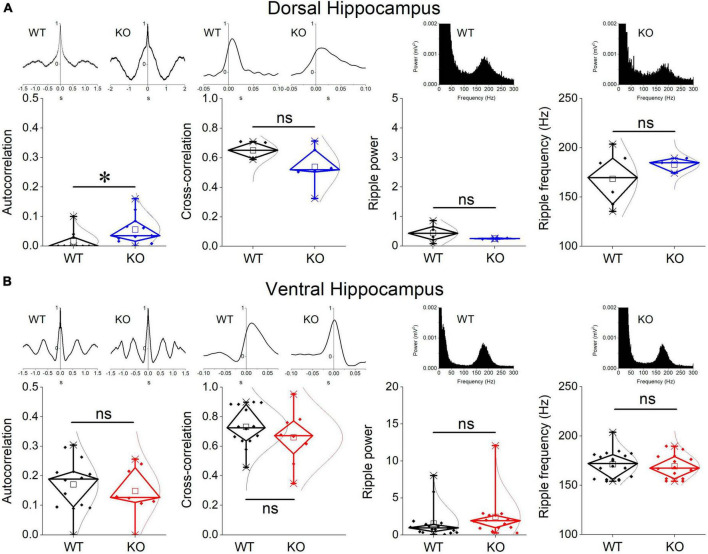
Comparison between WT and KO rats are shown for the CA1-CA1 autocorrelation, CA3-CA1 cross-correlation, and ripple oscillation in the dorsal **(A)** and ventral hippocampus **(B)**. Examples of autocorrelations, cross-correlations, and spectral power distributions are shown on the top of the main graphs. The results of statistical analysis (independent *t*-test) for the four variables are the following: autocorrelation: dorsal hippocampus *t*_29_ = –2.39, *p* = 0.024, WT = 11 rats, KO = 11 rats, and ventral hippocampus *t*_20_ = 0.58, *p* = 0.568, WT = 14 rats, KO = 8 rats; cross-correlation: dorsal hippocampus *t*_8_ = 1.51, *p* = 0.17, WT = 4 rats, KO = 6 rats, and ventral hippocampus *t*_20_ = 1.03, *p* = 0.316, WT = 14 rats, KO = 8 rats; ripple power: dorsal hippocampus *t*_5.135_ = 1.57, *p* = 0.175, WT = 6 rats, KO = 2 rats, and ventral hippocampus *t*_30_ = –1.023, *p* = 0.314, WT = 18 rats, KO = 14 rats; ripple frequency: dorsal hippocampus *t*_6.3_ = –1.18, *p* = 0.281, WT = 6 rats, KO = 3 rats, and ventral hippocampus *t*_30_ = 0.453, *p* = 0.654, WT = 18 rats, KO = 14 rats. Asterisk and “ns” denote statistically significant and not significant difference, respectively.

Events of SWRs occurred with significantly lower rate of occurrence, i.e., they displayed higher IEI in KO compared with WT dorsal hippocampus (Figure 2A). In contrast, IEI in the ventral KO hippocampus remained normal (Figure 2B). FXS did not significantly affect the amplitude of SWRs either in the dorsal or the ventral hippocampus. Furthermore, we observed no significant effect of genotype on the ability of the dorsal and ventral hippocampus to organize clusters of SWRs in general or clusters with more than 2 events (long clusters). In addition, ICI did not significantly change between WT and KO dorsal or ventral hippocampus. However, autocorrelation was significantly increased between WT and KO dorsal but not ventral hippocampus. Finally, no difference in CA3-CA1 cross-correlation was found between WT and KO dorsal or ventral hippocampus (Figure 3). The number of animals used, and the results of statistical analysis are shown in the corresponding figure legends. These results show that the activity of SWRs is altered in the dorsal but not the ventral hippocampus of KO rats in terms of frequency of occurrence and autocorrelation.

### 3.3 Ripples are not altered in the dorsal or ventral KO hippocampus

We measured ripple oscillation in the dorsal and ventral hippocampus and compared it between WT and KO rats. We found that the power of the oscillation did not significantly differ between WT and KO in either the dorsal (Figure 3A) or the ventral hippocampus (Figure 3B). Similarly, we found no change of the ripple frequency in the dorsal or ventral hippocampus between WT and KO rats (Figure 3). The results of statistical analysis are provided in the legend of Figure 3. These results suggested that ripple oscillation is not significantly affected in the hippocampus of KO compared with WT rats.

### 3.4 Reduced frequency of MUA-SWRs in the dorsal but not ventral KO hippocampus

**FIGURE 4:**
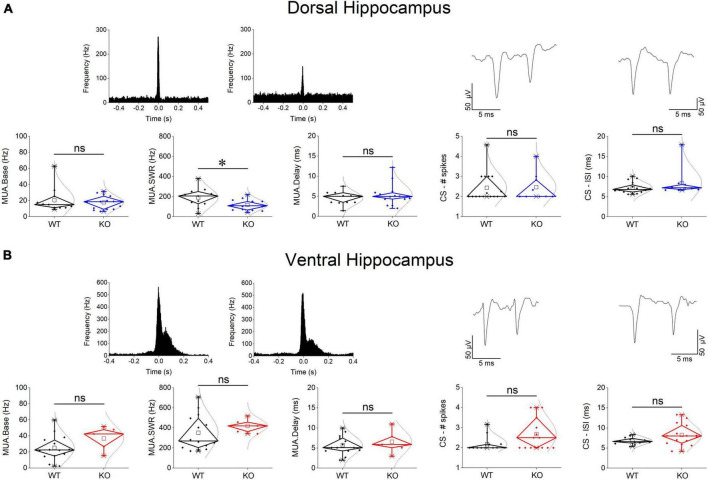
Genotype effects on MUA and CSB in the dorsal **(A)** and the ventral hippocampus **(B)**. Examples of MUA histograms triggered by the peaks of SPWs are shown on the top of MUA graphs. Examples of traces of CSB are shown on the top of the corresponding graphs of CSB. The results of statistical analysis (independent *t*-test) for the four variables are the following: MUA-Base: dorsal hippocampus *t*_23_ = 0.562, *p* = 0.58, WT = 11 rats, KO = 14 rats, and ventral hippocampus *t*_17_ = –1.43, *p* = 0.172, WT = 14 rats, KO = 5 rats; MUA-SWR: dorsal hippocampus *t*_23_ = 2.42, *p* = 0.024, WT = 11 rats, KO = 14 rats, and ventral hippocampus t–_16.37_ = –1.2, *p* = 0.247, WT = 14 rats, KO = 5 rats; MUA-Delay: dorsal hippocampus *t*_23_ = –0.884, *p* = 0.386, WT = 11 rats, KO = 14 rats, and ventral hippocampus *t*_17_ = –0.647, *p* = 0.526, WT = 14 rats, KO = 5 rats; CS-spikes: dorsal hippocampus *t*_23_ = –0.088, *p* = 0.931, WT = 16 rats, KO = 9 rats, and ventral hippocampus *t*_20.612_ = –2.07, *p* = 0.052, WT = 12 rats, KO = 15 rats; CS-ISI: dorsal hippocampus *t*_23_ = –1.224, *p* = 0.233, WT = 16 rats, KO = 9 rats, and ventral hippocampus *t*_18.05_ = –2.04, *p* = 0.056, WT = 12 rats, KO = 15 rats. Asterisk and “ns” denote statistically significant and not significant difference, respectively.

We assessed baseline multiunit activity (MUA-Base) as well as MUA occurring during events of SWRs (MUA-SWR). We found that the dorsal and ventral hippocampus of WT rats display similar frequency of MUA-Base (Table 1). However, the frequency of MUA-SWR was significantly higher in the ventral compared with the dorsal WT hippocampus (Table 1 and Figure 4). Genotype did not significantly affect the frequency of MUA-Base in either dorsal (Figure 4A) or ventral hippocampus (Figure 4B). However, we found that genotype significantly reduced the frequency of MUA-SWR in the dorsal KO vs. WT hippocampus; in contrast, MUA-SWR remained normal in the ventral KO hippocampus. The MUA-Delay was found similar in WT and KO dorsal (Figure 4A) and ventral hippocampus (Figure 4B). The corresponding results of statistical analysis are described in the legend of Figure 4. Interestingly, when all available data were pooled together, we observed that MUA-Base (Pearson correlation, *r* = 0.43, *p* = 0.002) but not MUA-SWR (Pearson correlation, *r* = 0.28, *p* = 0.052) positively and significantly correlated with the amplitude of SWRs. Also, the frequency of MUA-SWR was negatively and significantly correlated with the IEI (Pearson correlation, *r* = −0.362, *p* = 0.011), which corroborated the co-modulation of these parameters in the dorsal KO hippocampus.

### 3.5 Complex spike bursts (CSB) are normal in the KO hippocampus

Complex spikes bursts were observed in both segments of the hippocampus in WT and KO rats. We were able to detect 16 units in the dorsal and 12 units in the ventral WT hippocampus, and 9 units in the dorsal and 15 units in the ventral KO hippocampus. There was no significant difference in the number of spikes in CSB between the two hippocampal segments and we detected no dorso-ventral difference in ISI as well, either in WT or KO rats (see statistics in Tables 1, 2). Considering the genotype, we observed that the number of spikes and ISI were similar between WT and KO dorsal hippocampus (Figure 4A). Also, we observed that both the number of spikes and the ISI were higher in the ventral hippocampus of KO vs. WT rats, but not significantly so (Figure 4B). The fact that CSB activity remains unaffected in the KO hippocampus suggests that FXS does not impair a basic pattern of hippocampal pyramidal cells firing despite the plethora of changes produced by the loss of FMRP. See the legend of Figure 4 for results of statistical analysis.

**TABLE 2:** Comparisons of spontaneous activities between the dorsal and ventral hippocampus of KO rats.

		Dorsal hippocampus	Ventral hippocampus	Independent *t*-test
SWRs	Amplitude (μV)	70.9 ± 6.04 (24)	173.8 ± 27.31 (28)	*t*_29.63_ = −3.15, *p* = 0.001
	IEI (s)	2.29 ± 0.364 (24)	0.625 ± 0.052 (28)	*t*_23.95_ = 1.26, *p* < 0.001
	Probability clusters	0.128 ± 0.057 (8)	0.074 ± 0.024 (16)	*t*_22_ = 5.2, *p* = 0.308
	Probability long clusters	0.081 ± 0.053 (8)	0.066 ± 0.031 (16)	*t*_22_ = 3.22, *p* = 0.79
	ICI (ms)	101.4 ± 11.5 (6)	128.2 ± 7.5 (18)	*t*_22_ = −1.26, *p* = 0.079
	Auto-correlation CA1-CA1	0.056 ± 0.015 (11)	0.148 ± 0.03 (8)	*t*_17_ = −2.97, *p* = 0.009
	Cross-correlation CA3-CA1	0.539 ± 0.055 (6)	0.66 ± 0.066 (8)	*t*_12_ = −2.81, *p* = 0.205
Ripples	Power (mV × 10^–7^)	0.252 ± 0.014 (2)	2.44 ± 0.78 (14)	*t*_14_ = −1.64, *p* = 0.319
	Frequency (Hz)	182.9 ± 4.48 (3)	169.2 ± 3.2 (14)	*t*_15_ = −1.05, *p* = 0.08
MUA	MUA Baseline	17.7 ± 2.28 (14)	36.82 ± 6.57 (5)	*t*_4.92_ = −0.754, *p* = 0.046
	MUA–SWR	117.53 ± 14.38 (14)	420.51 ± 32.07 (5)	*t*_17_ = −2.93, *p* < 0.001
	MUA–Delay	5.34 ± 0.72 (14)	6.57 ± 1.35 (5)	*t*_17_ = −1.47, *p* = 0.406
CSB	No of spikes	2.46 ± 0.23 (9)	2.68 ± 0.21 (15)	*t*_22_ = −0.66, *p* = 0.516
	ISI (ms)	8.45 ± 1.2 (9)	8.27 ± 0.67 (15)	*t*_22_ = 0.146, *p* = 0.886

Values represent mean ± S.E.M.

In parenthesis is shown the number of rats used in the analysis for all variables except the variables of CSB where the number of units is shown.

### 3.6 Basal excitatory synaptic transmission

**FIGURE 5:**
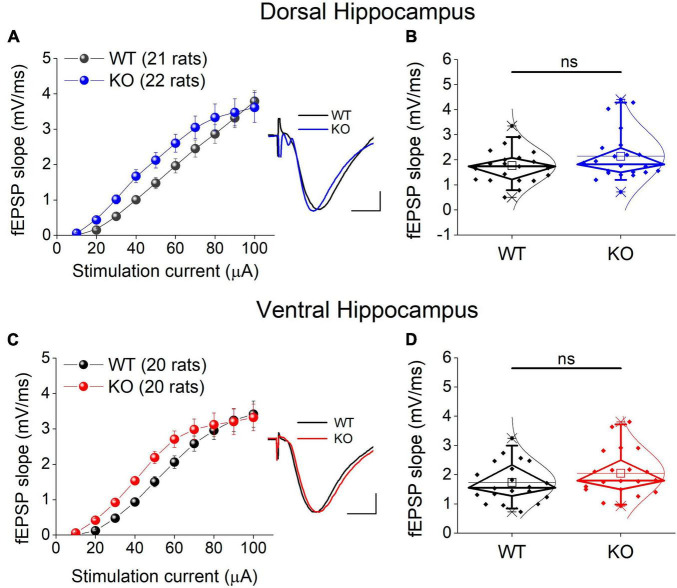
Excitatory synaptic transmission in the dorsal **(A,B)** and ventral KO hippocampus **(C,D)** compared between WT and KO rats. Input-output curves of fEPSP slope as a function of stimulation current intensity and box plots of the average fEPSP slope are shown on the left and right part of each panel, respectively. Example traces of fEPSP slopes are shown in inserts; calibration bars: 0.5 mV, 5 ms. Artifacts are truncated. Input-output curves from the dorsal hippocampus [UNIANOVA, *F*_(9,405)_ = 0.732, *p* = 0.679; WT = 21 rats, KO = 22 rats] and the ventral hippocampus [UNIANOVA, *F*_(9,376)_ = 0.82, *p* = 0.598; WT = 20 rats, KO = 20 rats] do not significantly differ between WT and KO rats. Similarly, the average fEPSP slope does not significantly differ between the two genotypes in either the dorsal (independent *t*-test, *t*_41_ = –1.42, *p* = 0.164; WT = 21 rats, KO = 22 rats) or the ventral hippocampus (independent *t*-test, *t*_38_ = –1.31, *p* = 0.198; WT = 20 rats, KO = 20 rats). “ns” denotes not significant difference.

We examined excitatory synaptic transmission by constructing input-output curves between stimulation current strength and fEPSP slope and calculating the average fEPSP slope from each curve. Comparing the entire input-output curves, we found no significant effect of genotype in either the dorsal or the ventral hippocampus (Figures 5A, C). Similarly, we found no significant effect of genotype on the average fEPSP slope in either segment of the hippocampus (Figures 5B, D).

### 3.7 Neuronal excitability

**FIGURE 6:**
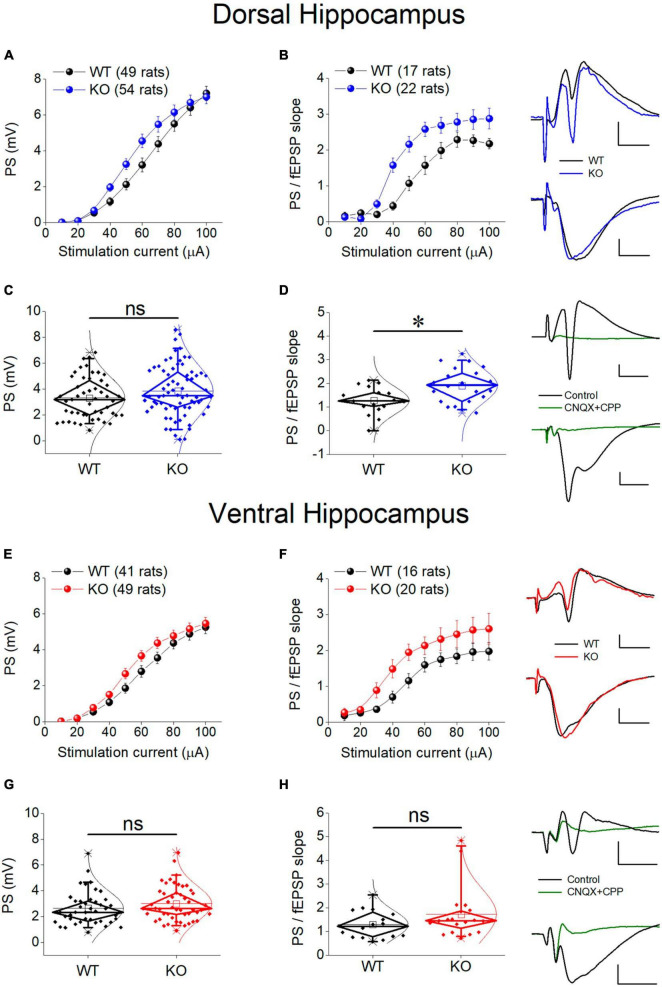
Neuronal excitability in the dorsal **(A–D)** and ventral **(E–H)** KO hippocampus compared with WT hippocampus. Collective input-output relationships of PS and PS/fEPSP slope for the dorsal and the ventral hippocampus are shown in **(A,B,E,F)**, respectively. The box plots for the average PS and PS/fEPSP slope of the dorsal and the ventral hippocampus are shown in **(C,D,G,H)**, respectively. Representative traces of simultaneously recorded fEPSP and PS are shown in the upper right of graphs of the Dorsal Hippocampus and Ventral hippocampus panels. Note that similar fEPSPs elicit higher PS in the KO compared with WT dorsal but not ventral hippocampus. Traces at the bottom right of the two panels illustrate that fEPSP and PS are abolished under blockade of AMPA and NMDA receptors by 40 μM CNQX and 10 μM CPP, respectively. Calibration bars: 0.5 mV, 5 ms. Artifacts are truncated. PS/I curve from the dorsal [UNIANOVA, *F*_(9,930)_ = 1.33, *p* = 0.216; WT = 49 rats, KO = 54 rats, **(A)**] and the ventral hippocampus [UNIANOVA, *F*_(9,812)_ = 0.66, *p* = 0.750; WT = 41 rats, KO = 49 rats, **(E)**] are similar in WT and KO rats; However, the PS/fEPSP slope curve is significantly shifted leftward in KO compared with WT dorsal hippocampus [UNIANOVA, *F*_(9,338)_ = 2.26, *p* = 0.018; WT = 17 rats, KO = 22 rats, **(B)**], but not ventral hippocampus [UNIANOVA, *F*_(9,317)_ = 0.353, *p* = 0.956; WT = 16 rats, KO = 20 rats, **(F)**]. The increased excitability in the KO vs. WT dorsal hippocampus is supported by the increased average value of the ratio PS/fEPSP slope [independent *t*-test, *t*_37_ = –2.91, *p* = 0.006; WT = 17 rats, KO = 22 rats, **(D)**]. However, in the ventral hippocampus, the average ratio PS/fEPSP slope is similar between WT and KO rats [independent *t*-test, *t*_34_ = –1.45, *p* = 0.155; WT = 16 rats, KO = 20 rats, **(H)**]. Also, the average PS value does not significantly differ between WT and KO dorsal [independent *t*-test, *t*_101_ = –1.45, *p* = 0.152; WT = 49 rats, KO = 54 rats, **(C)**] or ventral hippocampus [independent *t*-test, *t*_88_ = –1.26, *p* = 0.211; WT = 41 rats, KO = 49 rats, **(G)**]. Asterisk and “ns” denote statistically significant and not significant difference, respectively.

There is a growing consensus that cell and network excitability increases in several brain areas in individuals with FXS and *Fmr1*-KO rodents (Gibson et al., 2008; Qiu et al., 2009; Deng and Klyachko, 2016) including hippocampus (Chuang et al., 2005; Deng et al., 2019; Booker et al., 2020; Asiminas et al., 2022). However, studies in the hippocampus have been performed mainly in the dorsal segment of the structure and it is not yet known whether the ventral hippocampus responds to deficiency of FMRP similarly. Thus, we first examined whether the genotype affects the relationship between stimulation current strength and PS, and we found that the PS/I curve was significantly shifted leftward in KO compared with WT rat both in the dorsal and the ventral hippocampus (Figures 6A, E). However, comparing the average PS, we found that genotype did not significantly affect either the dorsal or the ventral hippocampus (Figures 6C, G).

An additional especially reliable index of postsynaptic excitability is the relationship between stimulation strength or postsynaptic depolarization and neuronal excitement. Thus, we assessed neuronal excitability by comparing I-O curves constructed by plotting PS/fEPSP slope ratio against stimulation current (excitability curves). We found that the excitability curve constructed from KO rats significantly shifted upward and leftward compared with WT rats in the dorsal but not the ventral hippocampus of the KO vs. WT rats (Figures 6B, F). We further examined excitability by comparing the average PS/fEPSP ratio and we found that it significantly increased in the dorsal but not the ventral hippocampus of KO vs. WT rat (Figures 6D, H). Both fEPSP slope and PS were abolished in the dorsal (*n* = 2 rats) and the ventral hippocampus (*n* = 2 rats) following application of 40 μM CNQX and 10 μM CPP in the perfusion medium (Figure 6), demonstrating that they are synaptically evoked events.

### 3.8 The expression of NMDARs is normal in the KO dorsal and ventral hippocampus

**FIGURE 7:**
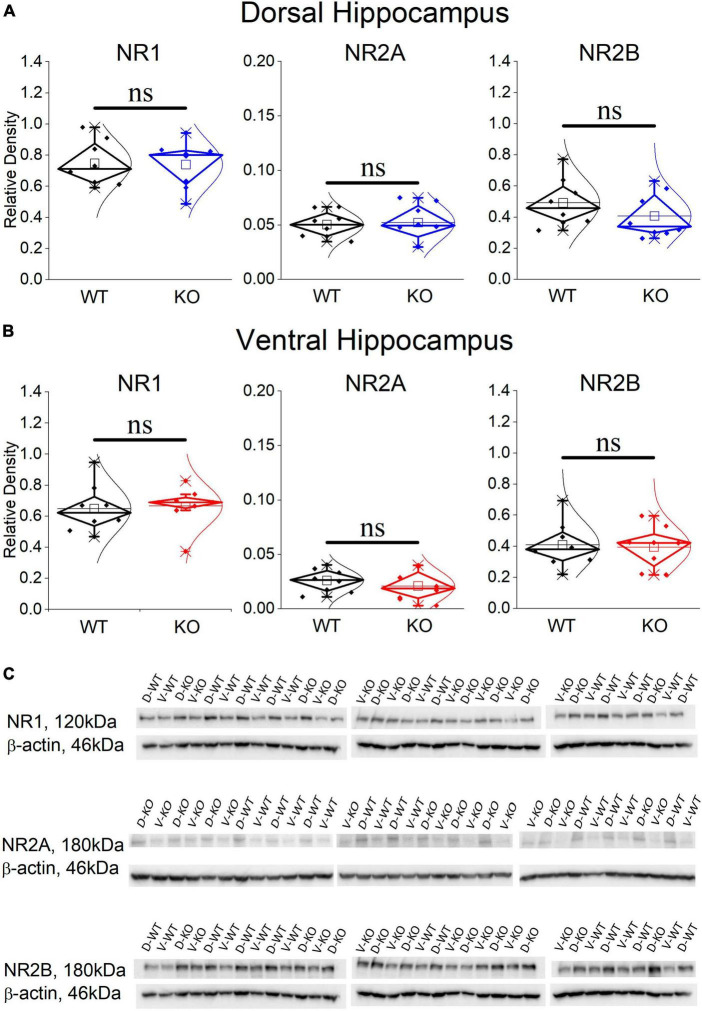
Western blotting analysis of the three NMDAR subunits, NR1, NR2A, and NR2B in WT and KO dorsal **(A)** and ventral hippocampus **(B)**. The results of the statistical analysis (independent *t*-test) showed similar expressions between the WT and KO dorsal hippocampus (NR1: t _14_ = 0.107, *p* = 0.813; NR2A: *t*_14_ = –0.24, *p* = 0.369; NR2B: *t*_14_ = 1.128, *p* = 0.842; *n* = 8 rats in either genotype), and ventral hippocampus (NR1: *t*_14_ = –0.241, *p* = 0.813; NR2A: *t*_14_ = 0.803, *p* = 0.435; NR2B: *t*_14_ = 0.203, *p* = 0.842; *n* = 8 rats in either genotype). Also, the NR2A/NR2B ratio is similar in WT and KO dorsal (*t*_10.15_ = –1.38, *p* = 0.199) and ventral hippocampus (*t*_9.72_ = 0.383, *p* = 0.71). The expression of NR2A subunit and the NR2A/NR2B ratio is higher in the dorsal compared with the ventral hippocampus in both WT (NR2A: *t*_14_ = 4.27, *p* < 0.001, and NR2A/NR2B ratio: *t*_14_ = 3.08, *p* = 0.008) and KO rats (NR2A: *t*_14_ = 4.017, *p* = 0.001, and NR2A/NR2B ratio: *t*_14_ = 3.01, *p* = 0.009). In contrast, NR1 and NR2B are similarly expressed in WT and KO dorsal (NR1: *t*_14_ = 1.308, *p* = 0.212, and NR2B: *t*_14_ = 1.09, *p* = 0.294) and ventral hippocampus (NR1: *t*_14_ = 1.039, *p* = 0.316, and NR2B: *t*_14_ = 0.187, *p* = 0.854). **(C)** Images of individual western blot samples with detected bands of the NMDA receptor protein subunits, and the corresponding loading marker band of beta actin. “ns” denotes not significant difference.

It has previously been shown that blockade of NMDARs reduces the incidence of SWRs (Papatheodoropoulos, 2010; Hunt et al., 2011; Kouvaros et al., 2015), the probability of clustered SWRs (Papatheodoropoulos, 2010; Kouvaros et al., 2015), and the neuronal firing during SWRs (Howe et al., 2020). Interestingly, the rate of SWRs and the frequency of firing activity during single events of SWRs (MUA-SWR) are reduced in the dorsal KO vs. WT hippocampus (see Figures 2, 4). Furthermore, the dorsal KO hippocampus shows a trend of reduction in the probability of clustered events (see Figure 2), and an increase in its network excitability, that has been previously shown to involve activation of NMDARs (Stasheff et al., 1993; Mangan and Kapur, 2004). Motivated by these observations, we wondered whether the expression of NMDARs is altered in the FXS hippocampus. Accordingly, we performed western blot experiments examining the expression of NR1, NR2A and NR2B subunits in the isolated CA1 region. Figure 7 shows that all three subunits of NMDARs are similarly expressed in WT and KO dorsal and ventral hippocampus. Comparing the NMDAR subunits between the two segments of the hippocampus we found increased levels of NR2A subunit as well as increased NR2A/NR2B ratio in the dorsal vs. ventral hippocampus in both genotypes, corroborating previous observations regarding the NR2A subunit and the NR2A/NR2B ratio (but not the NR2B subunit) in the WT Wistar rat (Pandis et al., 2006).

### 3.9 Inhibition increases in the ventral but not the dorsal KO hippocampus

**FIGURE 8:**
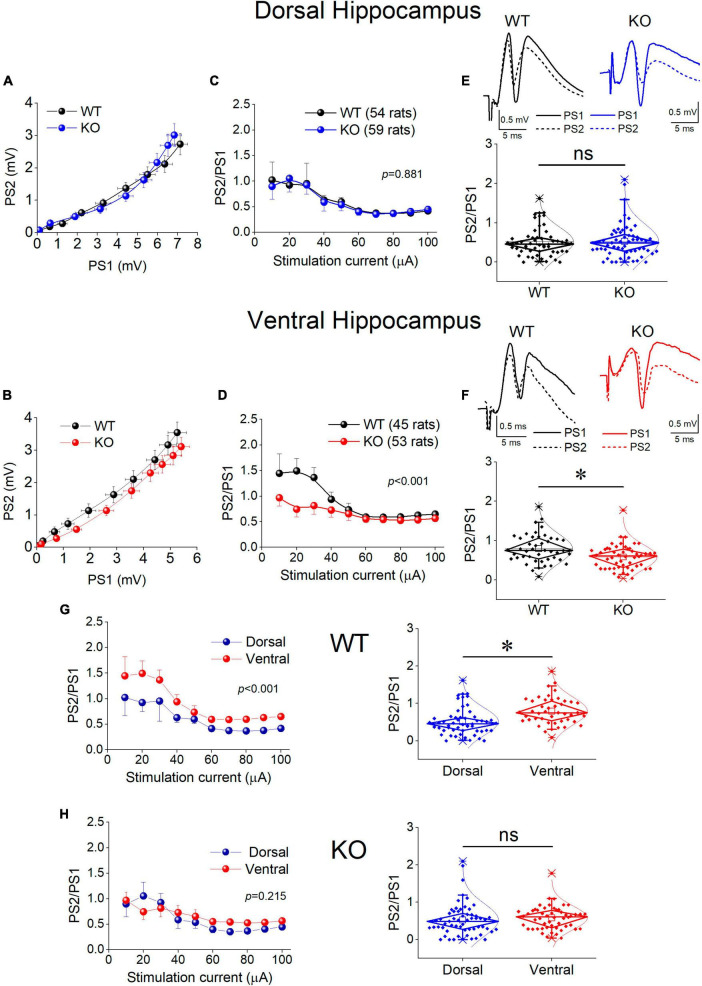
Inhibition increases in the KO ventral but not dorsal hippocampus. Inhibition was assessed using a paired-pulse stimulation protocol according to which the activation of principal neurons from the first pulse activates inhibitory cells that suppress firing of principal cells during the delivery of the second pulse (paired-pulse inhibition, PPI). Collective graphs between PS1 and PS2 for the dorsal and the ventral hippocampus are shown in **(A,B)**. Note that the curve obtained from the KO ventral hippocampus [UNIANOVA, *F*_(9,740)_ = 2.407, *p* = 0.011] is below that obtained from the WT ventral hippocampus, but this is not the case for the dorsal hippocampus [UNIANOVA, *F*_(9,827)_ = 0.126, *p* = 0.999]. Collective input-output curves of PS2/PS1 ratio plotted against stimulation current intensity are shown for the dorsal **(C)** and ventral hippocampus **(D)**. Box plots of average PS2/PS1 ratio for the dorsal and ventral hippocampus are shown in **(E,F)**, respectively. The average PS2/PS1 ratio is significantly lower in the KO compared with WT ventral hippocampus (independent *t*-test, *t*_95_ = 3.076, *p* = 0.003; WT = 45 rats, KO = 53 rats); however, PS2/PS1 ratio is similar in the WT and KO dorsal hippocampus (independent *t*-test, *t*_111_ = –0.169, *p* = 0.866; WT = 54 rats, KO = 59 rats). **(G,H)** The collective data are rearranged to illustrate that the significant difference in PPI between the dorsal and ventral hippocampus observed in WT rats (average PS2/PS1; independent *t*-test, *t*_97_ = –3.80, *p* = 0.001; dorsal hippocampus = 54 rats, ventral hippocampus = 45 rats) is eliminated in KO rats (independent *t*-test, *t*_110_ = –1.14, *p* = 0.257; dorsal hippocampus = 59 rats, ventral hippocampus = 53 rats). Asterisk and “ns” denote statistically significant and not significant difference, respectively.

Motivated by the observation that the activity of SWRs is normal in the ventral KO hippocampus despite an apparent increase in excitability, and the assumption that normal generation of SWRs requires a relatively well tuned balance between excitation and inhibition (E-I balance), we hypothesized that inhibition may be altered in the ventral hippocampus of KO rats. Inhibition of principal cell firing in the hippocampus is exerted by GABAergic interneurons which are activated by recurrent axonal collaterals of pyramidal neurons. A reliable method to assess the effectiveness of this inhibition to control local network excitation is the paradigm of paired-pulse stimulation where two identical presynaptic stimulations are applied in rapid succession and the activation of the effect of inhibitory local circuit which is activated by the first stimulation pulse is measured by the suppression of excitation produced by the second pulse. Thus, we compared the effectiveness of paired-pulse inhibition (PPI) between WT and KO rats by plotting the PS2/PS1 ratio as a function of stimulation current intensity (Figure 8).

We found that in the dorsal hippocampus neither the curves nor the average values of the PS2/PS1 ratio significantly differ between WT and KO rats (Figure 8, Dorsal Hippocampus). In contrast, we found a significant effect of genotype on PPI in the ventral hippocampus (Figure 8, Ventral Hippocampus). Specifically, the ventral hippocampus of KO vs. WT rats displayed a significantly smaller PS2/PS1 ratio and the corresponding curve was shifted toward smaller values. Strikingly, the robust dorsal-ventral difference in inhibition seen in WT rats and expressed by the input-output curves and the average PS2/PS1 ratio (Figure 8G, WT) was eliminated in KO rats as a result of the increased inhibition in the ventral KO hippocampus (Figure 8H). These results demonstrate a significant enhancement in the effectiveness of feedback inhibition in the ventral but not the dorsal hippocampus of KO compared with WT rats.

**FIGURE 9:**
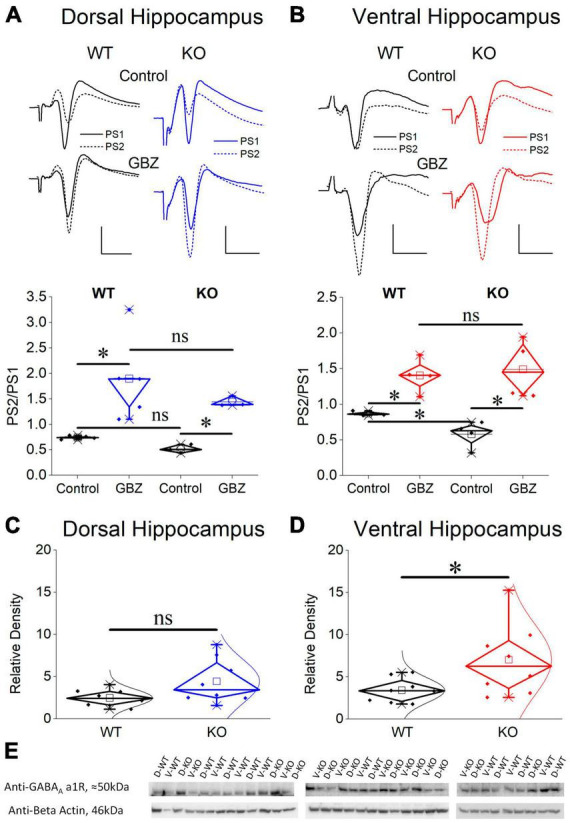
Blockade of GABA_*A*_ receptors eliminates the increased PPI in the KO ventral hippocampus which displays increased expression of GABA_*A*_ receptor α1 subunit compared with its WT counterpart. Application of gabazine (GBZ) for 10 min increases the PS2/PS1 ratio in both the dorsal hippocampus [**(A)**, independent *t*-test, WT: *t*_4_ = –3.09, *p* = 0.037, *n* = 5; KO: *t*_2_ = –9.01, *p* = 0.012, *n* = 3 slices] and the ventral hippocampus [**(B)**, independent *t*-test, WT: *t*_3_ = –5.01, *p* = 0.015, *n* = 4 slices; KO: *t*_3_ = –6.11, *p* = 0.009, *n* = 4 slices] of both genotypes. Furthermore, under gabazine the PS2/PS1 ratio is similar between WT and KO rats in both the dorsal (independent *t*-test, *t*_6_ = 0.911, *p* = 0.397, *n* = 3) and the ventral hippocampus (independent *t*-test, *t*_6_ = –0.365, *p* = 0.727, *n* = 4 slices), thus, eliminating the genotype-related difference in the ventral hippocampus. Normal distribution lines are omitted from these graphs for clarity. Example raw traces are shown on the top of the diagrams. Calibration bars: 1 mV, 5 ms. Expression of the α1 subunit of GABA_*A*_ receptors in the CA1 field of dorsal **(C)** and ventral hippocampus **(D)** from WT and KO rats. The ventral hippocampus displays increased expression of α1GABA_*A*_ receptors in KO compared with WT rats (independent *t*-test, *t*_8.593_ = –2.28, *p* = 0.048). In contrast, the expression of α1GABA_*A*_ receptors is similar in WT and KO dorsal hippocampus (independent *t*-test, *t*_8.993_ = –1.96, *p* = 0.082). At the bottom of figure are shown images of individual western blot samples with detected bands of the GABA_*A*_ receptor a1 protein subunit and the corresponding loading marker band of beta actin. **(E)** Images of individual western blot samples with detected bands of the GABAA receptor alpha 1 protein subunit, and the corresponding loading marker band of beta actin. Asterisk and “ns” denote statistically significant and not significant difference, respectively.

Considering that PS is shaped by both excitatory and inhibitory synaptic mechanisms and the excitatory component may contribute to defining PS2/PS1 ratio thereby affecting the genotype-dependent difference in PPI, we examined PS2/PS1 ratio under suppression of GABA_*A*_R-mediated inhibition by gabazine. Graphs in Figures 9A, B show that following application of gabazine the PS2/PS1 ratio increased in the dorsal and ventral hippocampus of both genotypes. Furthermore, blockade of GABA_*A*_Rs eliminated the difference in PPI between WT and KO ventral hippocampus suggesting that the excitatory component contributing to PS2/PS1 ratio is similar in WT and KO ventral hippocampus and the difference in PS2/PS1 ratio found under control conditions in WT and KO rats reflects difference in GABA_*A*_R-mediated inhibition.

### 3.10 Upregulation of α1GABA_*A*_R in the ventral but not the dorsal KO hippocampus

Considering the enhanced paired-pulse inhibition in the ventral KO hippocampus we questioned whether this increased effectiveness of the phasic inhibition is accompanied by a change in GABA_*A*_R expression. We chose to examine the alpha 1 (α1) subunit since α1 subunit containing GABA_*A*_R (α1GABA_*A*_R) is one of the most prevalent GABA_*A*_R subtypes in the brain including the hippocampal CA fields (Sieghart and Sperk, 2002). Furthermore, the presence of α1 subunit provides GABA_*A*_R with increased amplitude of inhibitory current (Vicini et al., 2001) and it could crucially contribute to enhanced inhibitory actions. The expression of α1 subunit in the dorsal CA1 hippocampal field did not significantly change between WT and KO rat (Figure 9C). Remarkably, however, we found an increased expression of α1 subunit in the ventral CA1 hippocampal field of the KO compared with WT rats (Figures 9D, E). These results corroborated the enhancement of PPI in KO ventral hippocampus, shown by the electrophysiological experiment.

### 3.11 Reduced Mg^2+^-free- induced epileptiform discharges in the ventral KO hippocampus

The interesting fact of the increased PPI in the ventral KO hippocampus, and the suggestion that the reduced inhibition in the ventral hippocampus (Petrides et al., 2007; Maggio and Segal, 2009; Milior et al., 2016) may represent a critical neurobiological background for the well-established increased susceptibility of the rodent ventral hippocampus (and the corresponding anterior hippocampus in human) to epileptic/epileptiform discharges (Burnham, 1975; Spencer et al., 1984; Gilbert et al., 1985; Bragdon et al., 1986; Lee et al., 1990; Greco et al., 1994; Akaike et al., 2001; Papatheodoropoulos et al., 2005; Haussler et al., 2012; Mikroulis and Psarropoulou, 2012; Moschovos et al., 2012), motivated us to hypothesize that the increased inhibition in the ventral hippocampus of KO rats may have an impact on its susceptibility to epileptiform discharges. Therefore, we examined whether the ventral hippocampus of KO rats could possibly display an increased resistance to epileptiform discharges. To test this hypothesis, we employed a common *in vitro* model of induction of interictal-like population discharges. Specifically, we perfused hippocampal slices with medium containing no magnesium ions (Mg^2+^-free medium) and we recorded population discharges. Spontaneous interictal-like discharges induced by Mg^2+^-free medium are thought to reflect large depolarizations produced mainly by activation of NMDARs (Dingledine et al., 1986) because of receptor relief from Mg^2+^-mediated blockade (Ascher and Nowak, 1988).

**FIGURE 10:**
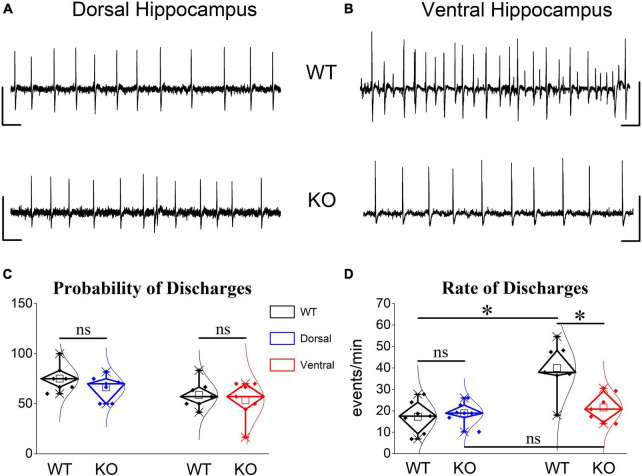
Epileptiform discharges induced after omission of Mg^2+^ from the perfusion medium are reduced in the ventral but not dorsal KO vs. WT hippocampus. Examples of interictal-like epileptiform discharges in the dorsal **(A)** and ventral hippocampus **(B)**. Calibration bars: 1 mV, 2 s. **(C)** Mg^2+^-free-induced discharges appeared with similar probability in dorsal and ventral hippocampus of WT (*n* = 5) and KO rats (*n* = 5). **(D)** The frequency of occurrence (rate) of epileptiform discharges does not differ between WT and KO dorsal hippocampus. However, the KO ventral hippocampus displayed a significantly reduced rate of epileptiform discharges compared with WT ventral hippocampus. The rate (*t*_12_ = –4.302, *p* = 0.001) but not the probability of appearance (*t*_12_ = 2.26, *p* = 0.05) is significantly higher in the ventral compared with the dorsal WT hippocampus (*n* = 7 rats, independent *t*-test). The rate of epileptiform discharges is similar between WT and KO dorsal hippocampus (independent *t*-test, *t*_12_ = –0.504, *p* = 0.624, WT = 7 rats and KO = 7 rats). In contrast, the rate of discharges is significantly lower in the KO vs. WT ventral hippocampus (independent *t*-test, *t*_12_ = 3.59, *p* = 0.004, WT = 7 rats and KO = 8 rats), eliminating the large dorsoventral difference that was initially observed in the WT rat (comparison between the two segments of the hippocampus of KO rat, independent *t*-test, *t*_12_ = –0.814, *p* = 0.432). Asterisk and “ns” denote statistically significant and not significant difference, respectively.

We observed that spontaneous large synchronous discharges resembling interictal discharges were spontaneously generated in dorsal and ventral slices from rats of both genotypes (Figures 10A, B). In WT rats, epileptiform discharges appeared in dorsal and ventral hippocampus with similar probabilities (Figure 10C). However, the frequency of occurrence (rate) of discharges was two times higher in the ventral (40.0 ± 4.48) compared with the dorsal hippocampus (17.27 ± 2.8) of the WT rat (Figure 10D). These results confirmed previous observations showing increasing rate of interictal-like epileptiform discharges along the dorsoventral axis of the rodent hippocampus see refs in Papatheodoropoulos (2018). Furthermore, the rate of discharges recorded from the dorsal hippocampus was similar between WT and KO rat (17.27 ± 2.8 vs. 18.97 ± 4.48) Figures 10A, D). Strikingly, the ventral hippocampus of the KO rat displayed a significantly reduced rate of discharges compared with the WT rat (21.52 ± 2.53 vs. 40.0 ± 4.48) (Figures 10B, D). Accordingly, the twofold dorsoventral difference in the rate of discharges observed in the WT rat disappeared in the KO rat. These results suggested that the increased inhibition in the ventral KO compared with WT hippocampus may contribute to reducing/limiting the tendency of the KO ventral hippocampus toward hyperexcitability.

### 3.12 Similar disinhibition-induced discharges in WT and KO hippocampus

**FIGURE 11:**
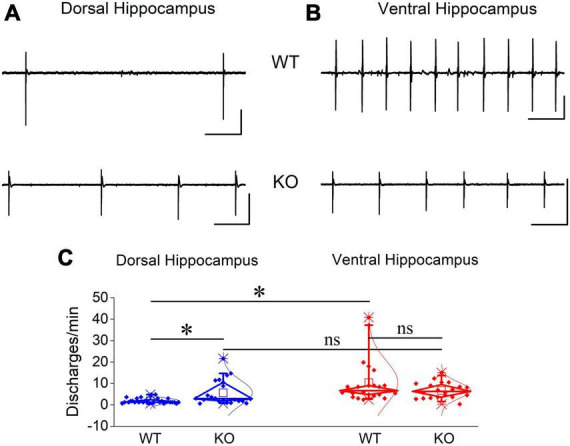
Blockade of GABA_*A*_ receptor-mediated transmission induced increased rate of epileptiform discharges in the dorsal but not ventral KO hippocampus. Examples of PTX-induced synchronized discharges in the dorsal **(A)** and ventral hippocampus **(B)**. Calibration bars: 1 mV, 10 s. **(C)** Discharges appeared with similar probability in dorsal and ventral hippocampus of WT (*t*_3_ = 1, *p* = 0.391, *n* = 4 rats) and KO rats (*t*_4_ = 0.344, *p* = 0.748, *n* = 3 rats; graph not shown). The rate of PTX-induced epileptiform discharges significantly increased in dorsal (independent *t*-test, *t*_22.4_ = –3.02, *p* = 0.006; *n* = 28 slices/4 WT rats and *n* = 22 slices/4 KO rats) but not ventral hippocampal slices (independent *t*-test, *t*_28.55_ = 1.61, *p* = 0.118; n = 23 slices/4 WT rats and *n* = 20 slices/4 KO rats) from KO compared with WT rats. Note that the rate of discharges is higher in the ventral (*n* = 23 slices/4 rats) than dorsal hippocampus (*n* = 28 slices/4 rats) (independent *t*-test, *t*_22.5_ = –4.02, *p* < 0.001) of WT rats.

Then, we hypothesized that blocking GABA_*A*_R-mediated inhibition could eliminate the region-specific genotype effect on population discharges. Perfusing hippocampal slices with PTX we observed that spontaneous population discharges appeared in almost all dorsal and ventral hippocampal slices from the WT (100 vs. 91.67 ± 8.3%) and KO rats (83.0 ± 11.33 vs. 76.67 ± 14.53%) (Figure 11). In WT rats the rate of PTX-induced population discharges was significantly higher in the ventral (10.4 ± 2.12 events/min) than dorsal hippocampus (1.84 ± 0.23 events/min), as also observed in the condition of Mg^2+^-free.

If the increased inhibition in the KO ventral hippocampus is involved in limiting the effect of its increased excitability and considering that network excitability increases in the KO vs. WT hippocampus, then, it could be expected that blockade of inhibition should provoke a similar worsening effect in both the dorsal and ventral KO vs. WT hippocampus. This was indeed what we observed in the dorsal hippocampus. PTX-induced discharges occurred with a significantly greater rate in the KO (5.65 ± 1.24 events/min) compared with the WT dorsal hippocampus (1.84 ± 0.23 events/min) (Figures 11A, C). The increased rate of epileptiform population discharges induced by PTX in the dorsal hippocampus is consistent with the increased network excitability we found with evoked responses in the KO hippocampus, i.e., PTX appears to uncover a greater amount of excitability in the KO compared with the WT dorsal hippocampus. Unexpectedly, however, the rate of PTX-induced discharges was similar between the WT (10.4 ± 2.12 events/min) and KO ventral hippocampus (6.73 ± 0.83 events/min) Figures 11B, C). These data are consistent with the idea that the increased inhibition in the KO ventral hippocampus limits its susceptibility to hyperexcitability.

## 4 Discussion

The main findings of the present study are the following: (a) the dorsal KO hippocampus displays reduced frequency of SWRs’ occurrence, reduced SWR-associated firing activity, and increased rhythmicity compared with the dorsal WT hippocampus; in contrast, the activity in the ventral hippocampus remains normal in KO rats; (b) local network excitability is enhanced in the dorsal but not the ventral hippocampus of KO rats; (c) synaptic inhibition and α1GABA_*A*_Rs are both upregulated in the ventral KO hippocampus only; and (d) the ventral, not dorsal, KO hippocampus is resistant to induced epileptiform discharges. This data represents the first comparative physiological study between dorsal and ventral hippocampus in an animal model of FXS.

### 4.1 Dorso-ventral differences in normal spontaneous activity

We show that SWRs and MUA significantly differ between the two segments of the hippocampus both in WT and KO rats. Specifically, in both WT and KO rats, the amplitude, and the autocorrelation of SWRs are significantly higher in the ventral compared with the dorsal hippocampus (Tables 1, 2). Some parameters differ between the two hippocampal segments only in WT or KO rats. In WT rats the ventral hippocampus displays a higher rate of SWRs’ occurrence while the dorsal hippocampus generates clusters of SWRs with increased probability compared with the ventral hippocampus (Table 1). Higher amplitude has been previously found in the ventral vs. dorsal hippocampus in WT Wistar rats (Kouvaros and Papatheodoropoulos, 2017). However, it is of note that the dorso-ventral difference in the probability of SWR’s clusters are opposite between the two rat strains. The reduction of the probability of clusters in the dorsal KO vs. WT hippocampus apparently eliminated the dorso-ventral difference in this variable seen in WT rats. Also, the higher MUA-Base observed in the ventral compared with the dorsal KO hippocampus, presumably reflects the combination between a moderate reduction of MUA-SWR in the KO dorsal hippocampus and a moderate enhancement in the ventral KO hippocampus.

### 4.2 FXS-associated effects on normal spontaneous activities

The main effect of FXS is to slow down SWRs and MUA-SWR only in the dorsal segment of the hippocampus. The apparent co-modulation of these two variables is highlighted by the observation that IEI and MUA-SWR are inversely correlated between each other. Ripples and CSB are not altered in the hippocampus of KO rats. In contrast to the dorsal hippocampus, SWRs remain normal in the ventral hippocampus.

Sharp wave-ripples are population events associated with intense, transient increase in neuronal excitability (Chrobak and Buzsáki, 1994). Moreover, the firing activity of the cell assembly that give rise to SWRs is highly organized representing specific spatiotemporal patterns of off-line re-activations of pyramidal cells, initially formed when the animal experiences an event (Wilson and McNaughton, 1994; Buzsaki, 2015; Foster, 2017). It has been shown that a dynamic and finely tuned balance between excitation and inhibition appears as required for a normal generation of SWRs (Buzsaki, 2015; Melonakos et al., 2019). Accordingly, changes in baseline network excitability may significantly influence the generation of SWRs, thereby affecting SWR-associated information processing (Hofer et al., 2015). Similarly, when excitation or inhibition is altered without a concomitant change of the other factor in this balance, a disturbance in the activity of SWRs may occur. For instance, relatively small to moderate enhancement of basal neuronal excitability following blockade/loss of potassium channels (Richter et al., 2008; Simeone et al., 2013; Trompoukis et al., 2020; Contreras et al., 2023) or elevation of extracellular potassium concentration (Papatheodoropoulos and Kostopoulos, 2002b), can greatly affect the pattern of SWRs’ generation.

The alterations in SWRs and MUA-SWR, we found in the dorsal KO hippocampus, were accompanied by an increase in the basal circuit excitability, as revealed by recordings of evoked potentials. The increased neuronal network excitability, often expressed as an increased E-I ratio, is a consistent observation in several brain regions of subjects with FXS including the hippocampus [see reviews by (Brager and Johnston, 2014; Contractor et al., 2015; Nelson and Valakh, 2015; Sohal and Rubenstein, 2019; Deng and Klyachko, 2021; Liu et al., 2021; Bülow et al., 2022)]. Several mechanisms may contribute to the increased excitability of the dorsal KO hippocampus, including dysregulation of various potassium channels such as Kv1, Kv4.2, large conductance potassium channels, A-type potassium channels, and hyperpolarization-activated cyclic nucleotide-gated channels (Gross et al., 2011; Brager et al., 2012; Routh et al., 2013; Zhang et al., 2014; Kalmbach et al., 2015; Brandalise et al., 2020; Kalmbach and Brager, 2020). These changes can importantly affect cellular properties leading to increased intrinsic and network excitability (Brager and Johnston, 2014). However, it is not obvious how an increased excitability is linked with a reduced frequency of SWRs and reduced SWR-associated firing activity. Synaptic influences do not appear to contribute to the increased network excitability of the KO dorsal hippocampus, as neither fEPSP slope nor expression of NMDARs are altered in the KO hippocampus.

The relationship between SWRs and baseline neuronal excitability is not necessarily linear in that optimal generation of SWRs may occur at intermediate levels of baseline excitability where excitation is balanced by inhibition, while insufficient or excessive levels in baseline excitability may disrupt normal organization of SPWs (Karlócai et al., 2014). Interestingly, balanced enhancement in excitation and inhibition that occur under normal conditions in the hippocampus *in vitro* appear to be beneficial for the generation of SWRs (Trompoukis et al., 2021). It is therefore conceivable to hypothesize that an enhancement of the network excitation in the dorsal KO hippocampus that is not accompanied by an analogous change in inhibition results in an increased network excitability and disturbance of excitation-inhibition balance that reduces the probability of occurrence of SWRs and disorganizes neuronal activity during SWRs, thereby affecting information processing in the dorsal hippocampus. In support of this idea is the recently reported observation that neuronal activity in the dorsal hippocampus of *Fmr1*-KO mouse during the performance of a spatial task is discoordinated (Radwan et al., 2016).

A basic component for normal generation of SWRs is GABA_*A*_R-mediated transmission. It has been established that SWRs require a highly organized activity of specific types of GABAergic interneurons (Somogyi et al., 2014). Specifically, parvalbumin-expressing (PV) basket cells increase their firing activity in synchrony with SWRs (Buzsaki et al., 1992; Klausberger et al., 2003) and optogenetic stimulation or silencing of PV GABAergic cells can trigger or inhibit the generation of SWRs, respectively (Schlingloff et al., 2014). A powerful phasic inhibition that limits excitation in hippocampal pyramidal cells (Andersen et al., 1964) occurs via activation of somatic GABA_*A*_Rs by PV basket cells (Freund and Buzsaki, 1996). Interestingly, SWR events correspond to fast GABA_*A*_R-mediated inhibitory postsynaptic potentials in CA1 pyramidal cells (Papatheodoropoulos and Kostopoulos, 2002a; Wu et al., 2002; Maier et al., 2003; Nimmrich et al., 2005; Papatheodoropoulos, 2008), and fast GABA_*A*_R-mediated currents in CA3 pyramidal cells represent a major component of the extracellularly recorded ripple oscillation (Schlingloff et al., 2014). Furthermore, relatively mild to moderate reductions in GABAergic transmission disrupts SWRs (Ellender et al., 2010; Liotta et al., 2011; Giannopoulos and Papatheodoropoulos, 2013; Karlócai et al., 2014). Accordingly, the increased inhibition in the ventral KO hippocampus may significantly support normal generation of SWRs in the ventral KO hippocampus. It is of note that soma targeting basket cells in the cerebellum of *Fmr1*-KO mice release more GABA because of a reduction of presynaptically located Kv1.1 potassium channels that results in enhanced excitability of the terminal and an increased Ca^2+^-dependent neurotransmitter release (Yang et al., 2020). It should be also noted that the absence of a change in network excitability observed in the ventral KO compared to the KO hippocampus could not rule out a change in neuronal intrinsic excitability as network excitability also depends on the level of inhibition, which is elevated in the ventral KO hippocampus. Thus, increased inhibition in the ventral KO hippocampus may act to reduce the effect of presumed increased neuronal excitability thereby endowing the local network with a balanced excitation/inhibition ratio and normal generation of SWRs.

On the other hand, the reduced rate of occurrence of SWRs in the KO vs. WT dorsal hippocampus might be related to a reduced functionality of PV cells, which can regulate the incidence of SWRs (Ellender et al., 2010). However, the number of PV interneurons has previously been found normal in the CA1 region of the KO dorsal hippocampus (Selby et al., 2007), and we did not find any significant genotype-related difference in PPI in the dorsal hippocampus. However, we cannot rule out that there is some change in the functionality of PV cells in the KO dorsal hippocampus affecting SWRs but not evoked responses. For instance, relatively small changes in GABAergic transmission, which do not affect PPI, can nevertheless reduce the rate of occurrence of SWRs (Giannopoulos and Papatheodoropoulos, 2013). In addition, a relatively mild reduction in GABA_*A*_R-mediated inhibitory potentials in the somatic area of pyramidal cells could reduce the incidence of SWRs by reducing post-inhibitory rebound excitation, which can trigger the generation of SWR event (Papatheodoropoulos, 2010).

Our finding of reduced rate of occurrence of SWRs in the dorsal KO vs. WT hippocampus is in keeping with previous observations made in mouse hippocampus *in vitro* (Pollali et al., 2021). We also confirm the absence of genotype effect on ripple oscillation reported by Pollali and coworkers (Pollali et al., 2021). However, it is of note that the FXS-associated alterations in SWRs in that study were obtained from the ventral-to-mid hippocampus of mice instead of the rat dorsal hippocampus we report here. In contrast to the dorsal hippocampus, we find normal SWRs in the rat ventral hippocampus. These seeming inconsistencies in the results obtained from rat and mice allow us to speculate that the region-specific effects of FXS on the hippocampus could be species-dependent, i.e., they might depend on the experimental animal model used. The fact that a previous *in vivo* study of SWRs performed in a mouse model of FXS reported no significant change in the rate of occurrence of SWRs in the dorsal hippocampus (Boone et al., 2018) suggests that a possible additional confounding factor might be related to the methodological approach used to study network oscillations. Interestingly, however, similarly to our results it has been previously reported that complex spike bursts in CA1 pyramidal neurons are normal in the *Fmr1*-KO mouse hippocampus (Boone et al., 2018; Ordemann et al., 2021). In any case, these data point to the need for a more systematic study of neuronal network activities in animal models of FXS.

### 4.3 FXS-associated effects on epileptiform discharges and a possible role of inhibition

A particularly interesting finding of the study is the combination of the preservation of normal SWR/MUA activity and the characteristic resistance to epileptic discharges exhibited by the ventral hippocampus of KO rats. The ventral hippocampus displays a constitutively increased network excitability that is suggested to represent a basic property of the ventral hippocampus network supporting its specific functional demands (Papatheodoropoulos, 2018). To a certain degree, the increased network excitability of the ventral hippocampus results from an increased intrinsic excitability of its principal cells (Maggio and Segal, 2009; Dougherty et al., 2012; Cembrowski et al., 2016) and its reduced phasic GABAergic inhibition (Petrides et al., 2007; Maggio and Segal, 2009; Milior et al., 2016). Though under normal conditions the ventral hippocampus functions effectively, however, under conditions that enhance network excitability it may cross the threshold to hyperexcitability resulting in the generation of epileptiform population discharges. Indeed, alongside its inherently increased excitability, the ventral hippocampus is the most susceptible brain region to epileptiform activity and seizures; see refs in Papatheodoropoulos (2018). Consequently, conditions that are accompanied by a heightened E-I ratio, such as FXS, could drive the ventral hippocampus toward a hyperexcitability state and associated aberrant activity that disrupts physiological information processing that causes behavioral deficits. Furthermore, the ventral hippocampus receives monosynaptic glutamatergic input from the basolateral nucleus of amygdala (Pikkarainen et al., 1999) the principal neurons of which are deficiently controlled by GABAergic inhibition in animal models of FXS (Olmos-Serrano et al., 2010). Consequently, the KO ventral hippocampus, by receiving a relatively increased excitation from amygdala faces an additional risk of hyperexcitability.

Individuals with FXS often display abnormalities in electroencephalogram and increased susceptibility to epilepsy (Kidd et al., 2014; Liu et al., 2021) with seizures occurring in about 12% of patients with FXS (Berry-Kravis et al., 2021). However, seizures occurring in children and teenagers with FXS usually disappear in adulthood (Sabaratnam et al., 2001; Kidd et al., 2014; Berry-Kravis et al., 2021), and seizures are rarely observed in adult patients suggesting that changes taking place during developing brain may ultimately reduce the likelihood of epileptic activity in the adulthood. Accordingly, we found that the ventral hippocampus from adult KO rats displays a striking resistance to epileptiform discharges, which display a greatly reduced rate in KO compared with the WT rats. A consequence is that the difference in susceptibility to epileptiform activity between the dorsal and the ventral hippocampus, one of the most established and prominent dorsoventral differentiations in the WT rat, disappears in the KO rat.

The ventral KO hippocampus, in addition to displaying reduced vulnerability to epileptic discharges compared with the WT counterpart, is apparently endowed with enhanced inhibition as suggested by the increased PPI and the augmented expression of α1GABA_*A*_Rs. It is noted that the α1 subunit, that is highly expressed in the CA1 hippocampal field (Sieghart and Sperk, 2002), confers a relatively increased amplitude of GABA_*A*_R-mediated inhibitory current (Vicini et al., 2001). Interestingly, elimination of α1 subunit is associated with increased susceptibility to seizures (Poulter et al., 1999; Kralic et al., 2002). Furthermore, upregulation of α1 protein subunit and GABAergic postsynaptic potentials have been observed in the hippocampus of mice with another neurodevelopmental disorder, namely neurofibromatosis type 1, which has a high prevalence of social deficits and autism (Costa et al., 2002; Gonçalves et al., 2017). Hence, an increased GABAergic inhibition suggested by the present evidence to occur in the ventral KO hippocampus could restrain local network excitation and prevent its transition into a state of hyperexcitability that might disrupt information processing. We therefore speculate that in addition to contributing to normal generation of SWRs, the maintenance of a dynamic E-I balance, by virtue of an increased inhibition, may assist the ventral KO hippocampus to stay away from a state of pathological excitability.

We should note that we found a reduced rate of discharges in the ventral KO hippocampus with the model of Mg^2+^-free medium but not the GABAergic disinhibition model. The Mg^2+^-free model allows for the examination of the role of GABAergic inhibition in induced population discharges (Perez Velazquez, 2003). Instead, eliminating the crucial factor of the inhibition (disinhibition model) the rate of epileptiform discharges becomes similar between WT and KO ventral hippocampus supporting the important role that inhibition may play in avoiding hyperexcitability in the ventral KO hippocampus. Dissimilar to the ventral hippocampus, the dorsal hippocampus of the KOs did not show any significant change in the rate of discharges in the Mg^2+^-free model compared with WT, presumably reflecting the absence of genotype-related change in inhibition. The dorsal KO hippocampus did, however, show an increased rate of epileptiform discharges in the disinhibition model. This seemingly results from the increased network excitability of the dorsal KO vs. WT hippocampus considering that the disinhibition model is suitable for examining the role of excitability in epilepsy (Meier and Dudek, 1996; Patrylo and Dudek, 1998). The fact that the rate of PTX-induced discharges did not increase in the ventral KO hippocampus may suggest that in addition to increased inhibition, other mechanisms may also contribute to restrict the proneness of the adult ventral KO hippocampus to epileptic activity.

## 5 Conclusion

In conclusion, the dorsal hippocampus of adult FXS rats shows altered SWRs and associated firing activity and an enhanced susceptibility to epileptiform discharges. In contrast, the ventral KO hippocampus, the segment of the structure with inherently increased excitability, displays normal SWRs and reduced susceptibility to epileptiform discharges, characteristics that are paralleled by an apparent upscaling of GABAergic inhibition. We propose that the neuronal network specifically in the ventral segment of the hippocampus is reorganized in adult *Fmr1*-KO rats by means of balanced changes between excitability and inhibition to ensure normal generation of SWRs and preventing at the same time derailment of the neural activity toward hyperexcitability.

## Data availability statement

The original contributions presented in the study are included in the article/supplementary material, further inquiries can be directed to the corresponding author.

## Ethics statement

The animal study was approved by (1) Protocol Evaluation Committee of the Department of Medicine of the University of Patras (2) Directorate of Veterinary Services of the Achaia Prefecture of Western Greece Region (reg. number: 5661/37, 18/01/2021). The study was conducted in accordance with the local legislation and institutional requirements.

## Author contributions

LL: Data curation, Formal analysis, Investigation, Writing – review and editing. GeT: Data curation, Formal analysis, Investigation, Writing – review and editing. GiT: Data curation, Formal analysis, Investigation, Writing – review and editing. AM: Data curation, Formal analysis, Investigation, Writing – review and editing. PF: Formal analysis, Investigation, Writing – review and editing. CP: Conceptualization, Data curation, Formal analysis, Funding acquisition, Methodology, Project administration, Supervision, Writing – original draft, Writing – review and editing.
